# Heart Rate Variability in Cardiometabolic Disease: From Molecular Mechanisms to Personalized Exercise Prescription

**DOI:** 10.3390/biomedicines14102221

**Published:** 2026-10-01

**Authors:** Héctor Fuentes-Barría, Raúl Aguilera-Eguía, Miguel Alarcón-Rivera, Cherie Flores-Fernández, Diego Ignacio Valenzuela Pérez, Lissé Angarita-Davila, Ángel Roco-Videla

**Affiliations:** 1 Centro de Investigación en Medicina de Altura (CEIMA), Universidad Arturo Prat, Iquique 1110939, Chile; 2 Departamento de Salud Pública, Facultad de Medicina, Universidad Católica de la Santísima Concepción, Concepcion 3349001, Chile; raguilerae@ucsc.cl; 3 Escuela de Ciencias del Deporte y Actividad Física, Facultad de Salud, Universidad Santo Tomás, Talca 3460000, Chile; mrivera3@santotomas.cl; 4 Departamento de Gestión de la Información, Universidad Tecnológica Metropolitana, Santiago 7550000, Chile; cflores@utem.cl; 5 Facultad de Salud y Ciencias Sociales, Universidad de las Americas, Santiago 8370003, Chile; dvalenzuelap@udla.cl; 6 Escuela de Nutrición y Dietética, Facultad de Medicina, Universidad Andres Bello, Concepción 3349001, Chile; lisse.angarita@unab.cl; 7 Dirección de Desarrollo y Postgrado, Universidad Autónoma de Chile, Santiago 7500912, Chile; angel.roco@uautonoma.cl

**Keywords:** heart rate determination, autonomic nervous system, cardiovascular diseases, exercise therapy

## Abstract

Cardiometabolic diseases are characterized by interconnected disturbances in metabolic, inflammatory, mitochondrial, vascular, endocrine, and autonomic regulation, highlighting the need for integrative biomarkers capable of capturing systemic physiological dysfunction. Heart rate variability (HRV) provides a non-invasive and dynamic measure of cardiovascular autonomic regulation and may reflect the cumulative physiological consequences of these interacting alterations. This narrative review synthesizes current evidence on the role of HRV in cardiometabolic disease, with particular emphasis on the molecular and physiological mechanisms linking autonomic dysfunction with metabolic, inflammatory, mitochondrial, and vascular disturbances, as well as its potential application in individualized exercise prescription. Across obesity, metabolic syndrome, insulin resistance, type 2 diabetes, hypertension, and cardiovascular disease, reduced HRV is consistently associated with impaired autonomic adaptability and increased cardiometabolic risk. However, HRV should not be interpreted as a direct measure of sympathovagal balance or as a molecular biomarker; rather, it represents an integrative physiological signal influenced by multiple biological, behavioral, and environmental factors. Exercise may modulate autonomic regulation while simultaneously influencing metabolic signaling, inflammation, mitochondrial function, vascular health, and recovery, and accumulating evidence indicates that improvements in HRV may accompany favorable adaptations across diverse cardiometabolic populations. Accordingly, repeated HRV assessment may complement clinical, metabolic, functional, and body-composition measures to characterize physiological adaptation and support individualized adjustments in exercise prescription. Overall, HRV represents a promising integrative marker of cardiometabolic health and physiological resilience. Nevertheless, methodological heterogeneity, physiological confounding, and limited causal evidence currently constrain its clinical implementation. Standardized longitudinal and mechanistic studies are warranted to establish clinically meaningful HRV phenotypes, determine their capacity to predict individual exercise responsiveness, and clarify whether HRV-guided interventions can ultimately improve cardiometabolic outcomes.

## 1. Introduction

Cardiometabolic diseases comprise a broad spectrum of interconnected conditions, including obesity, metabolic syndrome, insulin resistance, type 2 diabetes mellitus, hypertension, coronary artery disease, and heart failure [1,2]. Although these conditions are clinically heterogeneous, they share several biological mechanisms, including impaired glucose and lipid metabolism, adipose tissue dysfunction, endothelial impairment, chronic low-grade inflammation, oxidative stress, mitochondrial dysfunction, and dysregulation of neuroendocrine and autonomic pathways [3,4,5]. Rather than occurring independently, these mechanisms form a complex network in which alterations in one physiological system can amplify dysfunction in others [6].

The autonomic nervous system (ANS) occupies a central position within this network because it continuously integrates information originating from the cardiovascular, metabolic, immune, and endocrine systems [7]. Sympathetic and parasympathetic activity regulate heart rate, vascular tone, blood pressure, glucose metabolism, inflammatory responses, and energy expenditure [8,9,10]. Consequently, impaired autonomic regulation may represent both a consequence and a contributor to cardiometabolic disease progression [8].

Heart rate variability (HRV) refers to the physiological fluctuation in the time intervals between consecutive heartbeats [11]. Rather than representing random variability, these fluctuations arise from the continuous interaction among sympathetic and parasympathetic modulation, respiratory activity, baroreflex mechanisms, circulating hormones, metabolic signals, and central and peripheral neural inputs [12,13]. The classical Task Force recommendations established the methodological basis for HRV assessment and distinguished time-domain, frequency-domain, and geometric approaches [14]. More recent research has expanded HRV analysis to nonlinear and complexity-based measures, recognizing that cardiovascular regulation is inherently dynamic and multidimensional [15].

HRV has attracted increasing interest as a potential non-invasive biomarker of cardiometabolic health [16]. Reduced HRV has been reported in individuals with metabolic syndrome, obesity, insulin resistance, prediabetes, type 2 diabetes mellitus, hypertension, and cardiovascular disease [17,18,19]. A systematic review published by Stuckey et al. identified consistent associations between HRV alterations and several components of metabolic syndrome, although sex-specific differences and methodological heterogeneity were also observed [20]. More recently, a systematic review and meta-analysis of short-term HRV in metabolic syndrome demonstrated lower Standard Deviation of NN intervals (SDNN), Root Mean Square of the Successive Differences (RMSSD), low-frequency power, and high-frequency power compared with healthy controls [17]. These findings support the concept that autonomic dysfunction is closely associated with the metabolic phenotype.

Importantly, the relationship between HRV and cardiometabolic disease extends beyond simple autonomic imbalance [14]. Reduced HRV may reflect the cumulative influence of insulin resistance, systemic inflammation, oxidative stress, endothelial dysfunction, altered mitochondrial metabolism, and neuroendocrine activation [10,14,16]. In this context, HRV can be conceptualized as an integrative physiological signal that reflects the interaction among multiple biological systems rather than as an isolated surrogate of sympathetic or parasympathetic activity [14,21].

This perspective is particularly relevant to exercise medicine. Physical exercise induces acute and chronic changes in autonomic regulation, metabolic signaling, vascular function, inflammation, and mitochondrial biology [22,23]. Exercise training can increase vagally mediated HRV, improve autonomic recovery, and reduce cardiometabolic risk [24]. Evidence from type 2 diabetes mellitus populations indicates that exercise training can improve several HRV parameters, while broader meta-analytic evidence demonstrates beneficial effects of physical activity on HRV across populations [25,26,27].

Furthermore, HRV has emerged as a potential tool for individualized exercise prescription. HRV-guided training approaches use repeated measurements of autonomic modulation to adjust exercise intensity, volume, or training type according to individual physiological readiness [28,29,30]. Although evidence supporting HRV-guided training is stronger in endurance and athletic populations than in cardiometabolic disease, the underlying concept may be particularly relevant to patients with substantial inter-individual variability in metabolic and autonomic responsiveness [29,31].

Therefore, this narrative review aims to integrate current evidence regarding the role of HRV as a non-invasive physiological biomarker in cardiometabolic disease, with particular emphasis on the molecular and physiological mechanisms linking autonomic dysfunction with metabolic, inflammatory, mitochondrial, and vascular alterations. We further examine the effects of different exercise modalities on HRV and discuss how HRV may contribute to personalized exercise therapy and precision cardiometabolic medicine.

## 2. Literature Search Strategy

This narrative review was developed through a structured, non-systematic search of scientific literature addressing the relationships between HRV, cardiometabolic health, underlying molecular and physiological mechanisms, and exercise prescription. Literature searches were conducted in PubMed/MEDLINE, Scopus, and Web of Science, with additional relevant articles identified through the reference lists of retrieved publications and citation tracking.

The search strategy combined terms related to HRV with concepts representing cardiometabolic disease, autonomic regulation, metabolic dysfunction, inflammation, mitochondrial function, vascular function, and exercise. The principal search concepts included combinations of “heart rate variability,” “HRV,” “cardiometabolic disease,” “metabolic syndrome,” “obesity,” “insulin resistance,” “type 2 diabetes,” “hypertension,” “cardiovascular disease,” “autonomic dysfunction,” “inflammation,” “mitochondrial function,” “vascular function,” “exercise,” “exercise training,” “exercise prescription,” and “HRV-guided training.” Boolean operators (AND/OR) were used to combine these concepts as appropriate for each database.

The literature was examined from the earliest relevant publications available in the consulted databases up to the year August 2026, with greater emphasis placed on recent evidence and seminal studies that contributed substantially to the understanding of HRV in cardiometabolic disease. Articles were selected based on their relevance to the conceptual framework of the review, methodological contribution, clinical or physiological significance, and ability to support the mechanisms and applications discussed. Peer-reviewed original studies, systematic reviews, meta-analyses, consensus or methodological papers, and relevant clinical or physiological reviews were considered. Studies were not selected according to a formal systematic-review protocol, and no quantitative meta-analysis or formal risk-of-bias assessment was performed.

Because this was a narrative review, the literature selection was purposeful rather than exhaustive, allowing integration of evidence from complementary biological and clinical domains. Particular attention was given to studies that helped establish mechanistic links between autonomic regulation and metabolic, inflammatory, mitochondrial, vascular, and exercise-related adaptations. Additional publications were incorporated when they provided relevant methodological clarification regarding HRV measurement, interpretation, physiological confounding, or individualized exercise prescription. This approach was intended to provide a broad and integrative synthesis while maintaining transparency regarding the non-systematic nature of the selection of literature.

## 3. Physiological Basis of Heart Rate Variability

HRV represents the physiological variation in the time intervals between consecutive heartbeats, conventionally expressed as variations in successive RR intervals measured in milliseconds [11]. Although HRV is commonly described as a marker of autonomic nervous system activity, its physiological meaning is considerably broader [32]. The beat-to-beat fluctuations in cardiac rhythm emerge from the continuous interaction among autonomic neural regulation, respiratory activity, endocrine signaling, cardiovascular reflexes, hemodynamic conditions, metabolic demands, and intrinsic cardiac mechanisms [21,33,34]. Consequently, HRV can be conceptualized as a dynamic physiological biomarker that provides information regarding the capacity of the cardiovascular system to adapt to changing internal and external demands [21,35].

Traditionally, HRV has been assessed using electrocardiography (ECG), which provides accurate identification of successive R-wave peaks and therefore permits precise calculation of RR intervals [36]. However, technological advances have substantially expanded the accessibility of HRV monitoring. Smartphones, chest-worn sensors, smartwatches, and photoplethysmography-based wearable devices increasingly allow repeated assessment outside specialized laboratory settings [37,38,39]. Although photoplethysmography (PPG) based devices can provide reliable HRV measurements under resting conditions, their accuracy may be reduced during dynamic exercise because of motion artifacts, with ECG remaining the reference method for RR interval assessment during active movement [40]. This technological evolution has transformed HRV from a predominantly laboratory-based physiological measurement into a potentially longitudinal biomarker that can be assessed during daily life, exercise training, rehabilitation, sleep, and recovery [41].

The increasing accessibility of HRV monitoring is particularly relevant to cardiometabolic disease because these conditions are characterized by dynamic interactions among autonomic dysfunction, metabolic disturbances, inflammation, vascular impairment, and altered physiological recovery [16,18,21]. Persistent reductions in HRV have been associated with cardiovascular disease, hypertension, diabetes mellitus, metabolic syndrome, and obesity, suggesting that impaired autonomic adaptability may represent an early physiological manifestation of cardiometabolic dysregulation [17,18,19]. Importantly, HRV should not be interpreted as a direct molecular measurement. Rather, it represents an integrative physiological signal through which multiple molecular and systemic processes may become expressed at the level of cardiac autonomic regulation [35,42].

### 3.1. Cardiac Electrical Activity and Autonomic Regulation of Heart Rate

Physiological interpretation of HRV requires an understanding of the cardiac structures responsible for generating and propagating the electrical impulse. The heart is composed of the epicardium, myocardium, and endocardium and is surrounded by the pericardium, which protects the organ, limits excessive distension, and reduces friction associated with repetitive cardiac movement [43,44,45]. The myocardium is responsible for cardiac contraction, whereas the endocardium provides a smooth internal surface that facilitates blood flow. Functionally, the heart is organized into four chambers—two atria and two ventricles—that coordinate pulmonary and systemic circulation. The left ventricle generates the pressure required to maintain systemic perfusion, while the cardiac valves ensure unidirectional blood flow [43,44,45].

Cardiac contraction is coordinated by a specialized electrical conduction system composed principally of the sinoatrial (SA) node, atrioventricular node, bundle of His, and Purkinje fiber network. The SA node functions as the physiological pacemaker because of its spontaneous depolarization capacity and establishes the intrinsic rhythm of the heart. However, SA node activity is continuously modified by extrinsic regulatory systems, particularly the autonomic nervous system (ANS). This modulation explains why RR intervals are not perfectly constant and instead fluctuate from one heartbeat to the next, generating the physiological phenomenon measured as HRV [43,44,45].

The ANS provides rapid neural control of cardiac rhythm through the coordinated activity of the sympathetic nervous system (SNS) and parasympathetic nervous system (PNS). Sympathetic activation increases heart rate and myocardial contractility through the release of norepinephrine and activation of β-adrenergic receptors, thereby increasing cardiac output and oxygen delivery during physical or psychological stress [46,47]. In contrast, parasympathetic regulation is primarily mediated through the vagus nerve [12]. Acetylcholine released from vagal efferent terminals acts on muscarinic receptors within the sinoatrial node and reduces cardiac pacemaker activity, promoting energy conservation and facilitating recovery after physiological stress [47,48].

The balance between sympathetic and parasympathetic influences is dynamic rather than static [49]. During exercise or acute stress, sympathetic activation increases cardiac rate and contractility, whereas parasympathetic withdrawal contributes to the rapid elevation of heart rate [50]. Following cessation of exercise, reactivation of vagal activity represents one of the earliest mechanisms responsible for heart-rate recovery [12,51]. Thus, the instantaneous RR interval reflects the integrated activity of multiple regulatory systems rather than the isolated contribution of either autonomic branch [52].

Neuroendocrine mechanisms further contribute to cardiac regulation. Catecholamines released from the adrenal medulla enhance cardiovascular activation, while thyroid hormones influence metabolic rate and potentiate cardiovascular responses to physiological stress [53,54]. Cardiovascular reflexes provide an additional regulatory layer. Baroreceptors located primarily within the carotid sinus and aortic arch detect rapid changes in arterial pressure, whereas chemoreceptors respond to alterations in oxygen, carbon dioxide, and blood pH. These sensory systems continuously modify autonomic output to maintain cardiovascular homeostasis [55,56]. Local autoregulatory mechanisms also ensure that coronary blood flow remains matched to myocardial metabolic demand, particularly during exercise and conditions of increased cardiac workload [57].

Consequently, HRV represents the integrated output of a complex regulatory network involving the brain, autonomic nervous system, heart, vasculature, respiratory system, endocrine system, and metabolic tissues [12,35]. This systems-level interpretation is particularly important in cardiometabolic disease, in which autonomic dysfunction is closely interconnected with insulin resistance, chronic inflammation, endothelial dysfunction, oxidative stress, and altered metabolic regulation [33].

Under resting physiological conditions, healthy individuals generally exhibit substantial parasympathetic influence on cardiac rhythm [12,35]. This vagal predominance facilitates rapid beat-to-beat modulation and contributes to greater HRV [12,35]. Conversely, sustained sympathetic activation or reduced parasympathetic modulation can decrease HRV and has been described in obesity, metabolic syndrome, hypertension, diabetes mellitus, and cardiovascular disease [58]. Importantly, the reduction in HRV associated with these conditions should not be interpreted solely as a marker of autonomic imbalance [17,18,19]. Persistent sympathetic predominance may simultaneously promote inflammation, vascular dysfunction, altered glucose metabolism, and adverse cardiovascular remodeling, suggesting that autonomic dysregulation may participate in disease progression [59,60].

The relationship between autonomic regulation and cardiometabolic health is therefore bidirectional. Metabolic and inflammatory disturbances can impair autonomic control, while chronic autonomic dysregulation can influence vascular tone, glucose metabolism, inflammatory activity, and energy homeostasis [33,35]. This bidirectional interaction provides a physiological basis for considering HRV as an integrative biomarker of cardiometabolic function rather than merely a measure of cardiac autonomic tone [12,33,35].

### 3.2. Heart Rate Variability Domains and Physiological Interpretation

In clinical and research settings, HRV is traditionally characterized using three complementary analytical domains: time-domain, frequency-domain, and nonlinear methods. These approaches provide different perspectives on the temporal organization, oscillatory behavior, and complexity of cardiac autonomic regulation [42].

#### 3.2.1. Time-Domain Analysis

Time-domain methods directly quantify the variation between successive normal RR intervals [41]. Among the most commonly used indices are the SDNN and the RMSSD [61,62].

SDNN reflects overall RR interval variability within the recording period and therefore provides an estimate of global cardiac autonomic modulation. Because it is strongly dependent on recording duration, SDNN should be interpreted according to the temporal window used for assessment [42,61]. RMSSD, in contrast, emphasizes short-term beat-to-beat changes and is particularly sensitive to vagally mediated cardiac modulation. For this reason, RMSSD is widely used in exercise physiology and longitudinal monitoring of autonomic recovery [42,63,64].

The physiological relevance of RMSSD is particularly important for cardiometabolic populations. Persistent reductions in vagally mediated HRV may indicate diminished autonomic flexibility and impaired recovery capacity. Conversely, increases or preservation of RMSSD across repeated measurements may reflect improved parasympathetic modulation and enhanced physiological adaptability [65,66].

From a clinical monitoring perspective, RMSSD may currently represent one of the most practical HRV indices for repeated assessments of short-term autonomic modulation and recovery, particularly when measurements are obtained under standardized conditions. SDNN may provide complementary information regarding overall variability, but its strong dependence on recording duration makes direct interpretation of longitudinal changes more dependent on the measurement protocol [42,61]. Therefore, these parameters should not be considered clinically interchangeable, and the selection of an HRV index should be determined by the physiological question and recording duration.

Table 1 summarizes the main time-domain HRV measures.

#### 3.2.2. Frequency-Domain Analysis

Frequency-domain methods decompose RR interval variability according to oscillatory frequency bands, thereby providing information regarding the temporal organization of autonomic and cardiovascular fluctuations. The physiological interpretation of these components depends on recording duration, respiratory frequency, posture, and experimental conditions [67,68,69].

Very-low-frequency (VLF) oscillations are generally associated with slower regulatory processes, including thermoregulatory, metabolic, hormonal, and circadian influences. Low-frequency (LF) variability reflects a combination of autonomic and cardiovascular regulatory mechanisms, including baroreflex-related processes. High-frequency (HF) variability is closely related to respiratory sinus arrhythmia and is strongly influenced by parasympathetic modulation [42,67,68,69].

The duration of HRV recordings also determines the physiological information that can be obtained. Ultra-short recordings can characterize rapid autonomic responses, whereas short-term recordings provide information regarding resting autonomic regulation under standardized conditions. Longer recordings extend the assessment to circadian, hormonal, behavioral, and environmental influences [42,70,71,72]. Therefore, HRV should always be interpreted in relation to the recording duration and physiological context.

Importantly, the LF/HF ratio should be interpreted cautiously. Although historically used as an indicator of “sympathovagal balance,” contemporary physiological interpretations recognize that LF and HF components are influenced by multiple overlapping mechanisms and cannot be considered direct measures of sympathetic and parasympathetic activity, respectively. Consequently, a comprehensive assessment should prioritize physiologically validated indices and consider multiple HRV parameters rather than relying on a single ratio [42,67].

For clinical and longitudinal monitoring, HF-related measures may provide complementary information regarding vagally mediated and respiratory-related modulation, particularly when respiratory conditions are adequately controlled. However, their interpretation is sensitive to breathing frequency and other experimental conditions [67,68,69]. Accordingly, frequency-domain indices may be most informative when used to complement time-domain measures rather than as isolated clinical markers. In particular, LF/HF should not be used as a primary indicator of sympathovagal balance or as a stand-alone measure for clinical decision-making [42,67].

Table 2 summarizes the main frequency domain HRV measures.

#### 3.2.3. Nonlinear and Complexity-Based Analysis

The cardiovascular system is a complex biological system characterized by nonlinear interactions across multiple temporal scales. Accordingly, nonlinear HRV methods complement conventional time- and frequency-domain measures by quantifying the organization and complexity of cardiac dynamics [42,67].

Common approaches include Poincaré plots, approximate entropy (ApEn), sample entropy (SampEn), detrended fluctuation analysis (DFA), and fractal-dimension measures. Poincaré analysis provides information regarding short- and long-term beat-to-beat variability, whereas entropy-based measures quantify the regularity and complexity of the RR interval series. DFA assesses fractal scaling properties and can identify changes in the organization of cardiac dynamics that may not be captured by conventional linear measures [42,73,74,75].

This expanded analytical perspective is particularly relevant to cardiometabolic disease because pathological states may not simply reduce the magnitude of HRV but may also alter the complexity and adaptability of cardiovascular regulation [12,33,35]. Thus, nonlinear measures may provide complementary information regarding the loss of physiological complexity associated with metabolic and cardiovascular dysfunction [76,77].

Collectively, the three analytical domains support the interpretation of HRV as a multidimensional biomarker [12,35]. Time-domain indices quantify variability magnitude, frequency-domain methods characterize oscillatory regulation, and nonlinear measures provide information regarding complexity and adaptability [42,67]. The integration of these approaches may therefore provide a more comprehensive representation of cardiovascular autonomic function than any individual HRV parameter [78].

Nevertheless, the broader range of nonlinear measures should not be interpreted as evidence that all HRV parameters have equivalent clinical utility. For routine longitudinal monitoring, time-domain measures—particularly RMSSD under standardized short-term conditions—currently offer a relatively practical and reproducible approach to assessing short-term autonomic modulation and recovery [42,63,64]. SDNN may provide complementary information when recording duration is consistent, whereas frequency-domain and nonlinear indices may be used to provide additional physiological information when the recording protocol and analytical conditions are appropriately standardized [42,67]. Nonlinear measures such as DFA and entropy indices remain valuable primarily as complementary measures of cardiovascular complexity and adaptability, but their clinical interpretation and implementation require further standardization [73,74,75].

Thus, a practical hierarchy may be considered for future clinical monitoring: RMSSD as a primary short-term index of vagally mediated modulation; SDNN as a complementary measure of overall variability when recording duration is standardized; selected frequency-domain measures as contextual or complementary indices; and nonlinear measures as additional markers of complexity and adaptability rather than routine stand-alone clinical metrics. This hierarchy is intended to guide interpretation rather than imply that a single HRV parameter is sufficient to characterize autonomic function [42,67,78].

Table 3 summarizes the main non-linear domain HRV measures.

## 4. Cardiometabolic Dysregulation and HRV

Cardiometabolic disease is not the consequence of a single pathological pathway but rather the result of interconnected disturbances involving adipose tissue, glucose and lipid metabolism, inflammation, oxidative stress, mitochondrial function, vascular homeostasis, and neuroendocrine regulation [3,4,5,6]. These processes converge on cardiovascular control and progressively impair the capacity of the organism to adapt to changes in metabolic and physiological demand [33]. Within this complex network, HRV may provide a non-invasive representation of the integrated consequences of cardiometabolic dysfunction on cardiovascular autonomic regulation [17,42,67,79]. Importantly, HRV should not be interpreted simply as a surrogate of a binary sympathetic–parasympathetic balance [67,80]. Rather, it reflects the dynamic interaction between cardiac autonomic modulation and multiple metabolic, vascular, respiratory, endocrine, inflammatory, and environmental influences [12,35,81,82].

The association between cardiometabolic dysfunction and reduced HRV has been described across obesity, metabolic syndrome, insulin resistance, type 2 diabetes mellitus, hypertension, and cardiovascular disease [17,18,19]. However, the magnitude and clinical expression of autonomic alterations vary considerably among individuals, even when conventional indicators such as body mass index (BMI), blood glucose, or blood pressure are similar [83]. This heterogeneity suggests that HRV may capture an additional dimension of cardiometabolic phenotype that is not fully represented by conventional clinical measurements [84,85]. Such variability is influenced by age, sex, physical fitness, medication, lifestyle, sleep, psychological stress, and methodological characteristics of HRV assessment [67].

A mechanistic interpretation of this relationship requires considering cardiometabolic dysfunction as a network rather than a sequence of independent abnormalities [33]. Adipose-tissue dysfunction may promote inflammation and insulin resistance; insulin resistance and ectopic lipid accumulation may increase oxidative and mitochondrial stress; these alterations may impair endothelial and vascular function; and persistent metabolic and inflammatory signaling may modify neuroendocrine and autonomic regulation [86,87,88]. Conversely, chronic autonomic dysregulation may contribute to altered glucose metabolism, vascular function, inflammatory signaling, and energy homeostasis [89]. Thus, the relationship between cardiometabolic disease and HRV is potentially bidirectional, creating a feedback loop that progressively reduces physiological resilience [12,35].

**Figure 1:**
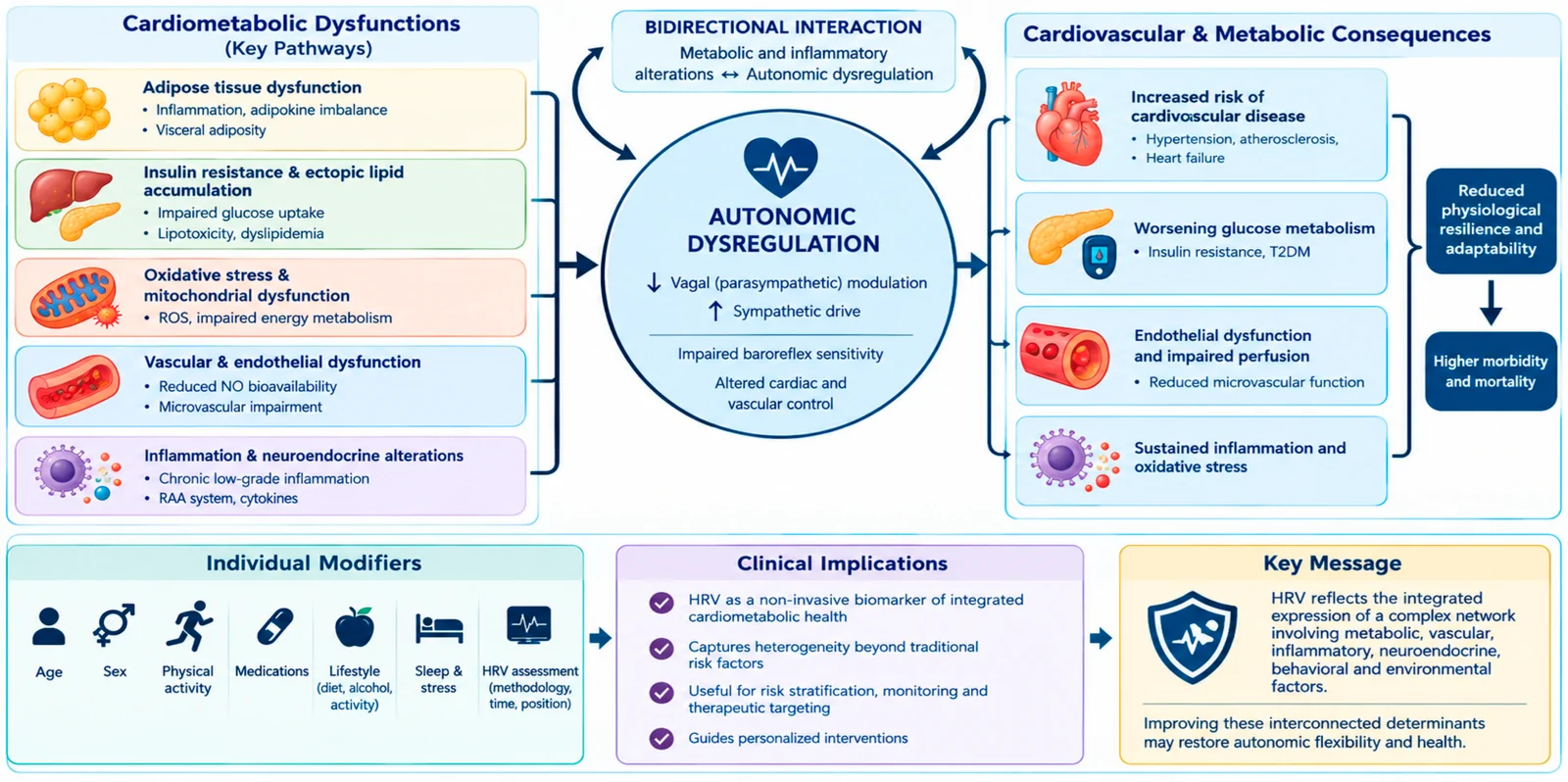
Cardiometabolic Dysregulation and HRV. Source: Elaboration owns.

Figure 1 illustrates the proposed bidirectional relationship between cardiometabolic dysfunction and autonomic nervous system dysregulation. Multiple interconnected cardiometabolic pathways—including adipose tissue dysfunction, insulin resistance and ectopic lipid accumulation, oxidative stress and mitochondrial dysfunction, vascular and endothelial impairment, and chronic low-grade inflammation with neuroendocrine alterations—contribute to autonomic imbalance. This imbalance is characterized by reduced HRV, impaired parasympathetic (vagal) modulation, increased sympathetic activity, altered baroreflex sensitivity, and disturbed cardiac and vascular autonomic control. Among these neuroendocrine pathways, hyperactivity of the renin–angiotensin–aldosterone system (RAAS) may further contribute to autonomic dysregulation by enhancing sympathetic drive through angiotensin II–mediated central and peripheral mechanisms. Increased RAAS activity may therefore reinforce sympathetic activation, impair parasympathetic modulation, and contribute to reduced HRV in cardiometabolic disease. Conversely, autonomic dysregulation may further exacerbate metabolic and inflammatory disturbances, establishing a self-perpetuating bidirectional cycle.

The resulting autonomic impairment is associated with several major cardiovascular and metabolic consequences, including an increased risk of cardiovascular disease (e.g., hypertension, atherosclerosis, and heart failure), worsening glucose metabolism and insulin resistance, endothelial dysfunction and impaired microvascular function, and sustained inflammation and oxidative stress. Collectively, these processes may reduce physiological resilience and adaptability, ultimately contributing to increased morbidity and mortality.

The lower panel highlights individual modifiers that may influence autonomic function and HRV, including age, sex, physical activity, medication use, lifestyle factors, sleep and stress patterns, and the conditions under which HRV is assessed (e.g., methodology, body position, and time of measurement). These factors should be considered when interpreting HRV as a marker of integrated cardiometabolic health. From a clinical perspective, HRV provides a non-invasive measure of autonomic regulation that may help capture heterogeneity beyond conventional risk factors and support risk stratification, longitudinal monitoring, and the identification of potential therapeutic targets. Overall, the figure emphasizes that HRV reflects the integrated expression of a complex network involving metabolic, vascular, inflammatory, neuroendocrine, behavioral, and environmental influences, and that improving these interconnected determinants may contribute to restoring autonomic flexibility and physiological resilience.

### 4.1. Adipose Tissue Dysfunction and Autonomic Remodeling

Obesity provides a prototypical example of the interaction between metabolic dysfunction and autonomic regulation [90]. Contemporary definitions of obesity increasingly recognize that excess body mass alone does not adequately characterize the biological consequences of adiposity [91,92,93]. Obesity involves alterations in endocrine, inflammatory, mitochondrial, metabolic, and cardiovascular regulation, and individuals with similar BMI may exhibit markedly different distributions of adipose and lean tissue and, consequently, different cardiometabolic risk profiles [94,95,96].

This distinction is particularly relevant to HRV because adipose tissue is an active endocrine and immunometabolic organ [97,98]. It releases adipokines, cytokines, free fatty acids, and other bioactive mediators that influence glucose metabolism, inflammation, vascular function, and neural regulation [99,100]. Visceral adipose tissue appears particularly important because its expansion is associated with increased lipolysis, insulin resistance, dyslipidemia, endothelial dysfunction, and chronic inflammation. Visceral adipose expansion also promotes immune-cell infiltration, including proinflammatory macrophage accumulation, increased reactive oxygen species generation, and disruption of intracellular metabolic signaling [101,102,103,104].

These alterations provide several potential pathways through which adipose dysfunction may influence cardiac autonomic regulation [33]. Chronic inflammatory and metabolic signaling can modify central autonomic networks, peripheral neural pathways, vascular regulation, and cardiac responsiveness [105]. In this context, reduced vagally mediated HRV may reflect a loss of autonomic flexibility accompanying metabolic dysfunction rather than an isolated abnormality of cardiac control [106,107]. Although obesity is frequently characterized by reduced parasympathetic modulation and relative sympathetic predominance, this pattern should not be reduced to a simplistic “sympathetic versus parasympathetic” model [33,108]. Instead, altered HRV may represent the integrated consequence of adipose dysfunction, inflammation, insulin resistance, oxidative stress, endothelial impairment, and neuroendocrine alterations [12,35].

Body composition may therefore provide important context for interpreting HRV [14]. Skeletal muscle is a major site of glucose disposal, substrate oxidation, energy expenditure, and myokine secretion, whereas adipose tissue contributes substantially to endocrine and inflammatory signaling [109]. Consequently, the relative distribution of fat and lean mass may influence both metabolic health and cardiovascular autonomic regulation [110].

This perspective challenges the use of body weight or BMI as the sole descriptors of cardiometabolic phenotype [33,83,111]. Greater visceral adiposity combined with reduced skeletal-muscle mass, commonly referred to as sarcopenic obesity, may represent a particularly unfavorable metabolic and autonomic phenotype. Conversely, reductions in adiposity accompanied by preservation or enhancement of lean mass may produce more favorable metabolic conditions without necessarily generating large changes in total body weight [101,102]. Therefore, the integration of body composition and HRV may provide a more informative characterization of cardiometabolic status than either measure alone [112].

### 4.2. Insulin Resistance and Neurocardiometabolic Signaling

Insulin resistance represents a central mechanistic link between adipose dysfunction and cardiometabolic disease [113]. Excess visceral adiposity, increased circulating free fatty acids, inflammation, and ectopic lipid accumulation interfere with insulin signaling and progressively impair the capacity of peripheral tissues to appropriately regulate glucose metabolism [114,115]. Skeletal muscle is particularly important because it represents a major site of insulin-stimulated glucose disposal; consequently, reduced muscle mass or impaired muscle quality may further contribute to systemic metabolic dysfunction [103].

The consequences of insulin resistance extend beyond glucose homeostasis. Altered insulin signaling can influence sympathetic activity, vascular tone, substrate availability, and central nervous system regulation [116]. In particular, compensatory hyperinsulinemia associated with insulin resistance may stimulate central sympathetic outflow through hypothalamic pathways, providing an important mechanism linking insulin resistance to increased sympathetic activity and reduced HRV [117]. At the same time, insulin resistance frequently coexists with inflammation, oxidative stress, endothelial dysfunction, and mitochondrial impairment [118]. These processes provide multiple converging pathways through which metabolic dysfunction may impair cardiovascular autonomic regulation [119].

The relationship is also potentially bidirectional. Autonomic regulation influences glucose metabolism, vascular function, energy expenditure, and endocrine signaling, whereas persistent metabolic dysfunction may alter autonomic activity and reduce cardiovascular adaptability [120,121]. Accordingly, HRV may provide an integrated physiological signal of the interaction between metabolic status and neurocardiovascular regulation rather than simply reflecting cardiac autonomic tone [12,35,122].

This interpretation may be particularly relevant across the spectrum extending from obesity and metabolic syndrome to prediabetes and type 2 diabetes [17,20,79,123,124]. Autonomic abnormalities may emerge alongside metabolic dysfunction before advanced cardiovascular complications become clinically evident [11,125]. However, HRV should be regarded as a complementary physiological biomarker rather than a direct measurement of insulin resistance or a replacement for established metabolic assessments [11,12,35].

### 4.3. Inflammation, Oxidative Stress, and the Autonomic–Immune Interface

Chronic low-grade inflammation represents a fundamental feature of cardiometabolic dysfunction and provides an important mechanistic bridge between metabolic abnormalities and autonomic regulation [126,127]. Expansion of adipose tissue promotes a proinflammatory secretory phenotype characterized by increased production of inflammatory mediators, reactive oxygen species, and other signaling molecules [110,128]. These alterations may interfere with insulin signaling, lipid metabolism, endothelial function, and neurovegetative regulation [126,127,128].

The interaction between inflammatory signaling and autonomic regulation is reciprocal. Persistent inflammatory activation may influence central autonomic networks and peripheral cardiovascular signaling, while altered autonomic regulation may in turn modify inflammatory responses [129]. A key mechanism underlying this interaction is the cholinergic anti-inflammatory pathway (CAP), through which vagal efferent activity can modulate systemic inflammatory responses by activating α7 nicotinic acetylcholine receptors (α7nAChR) expressed on immune cells, thereby attenuating the release of proinflammatory cytokines [130]. This bidirectional relationship provides a physiological basis for considering HRV as an indirect marker of the autonomic–immune interface. Reduced vagally mediated HRV in metabolically unhealthy individuals may therefore reflect a broader loss of physiological adaptability rather than an isolated cardiac abnormality [11,12,35].

Oxidative stress further strengthens this interaction. Excessive reactive oxygen species generation can impair intracellular signaling, endothelial function, mitochondrial metabolism, and vascular homeostasis [131]. In the context of obesity and insulin resistance, increased oxidative stress may therefore amplify both metabolic and autonomic dysfunction [132,133]. Importantly, these mechanisms should be considered interconnected rather than independent: inflammation can increase oxidative stress, oxidative stress can impair insulin and endothelial signaling, and both processes can contribute to autonomic dysregulation [126,127].

From this perspective, HRV may be interpreted as a physiological endpoint emerging from the interaction of metabolic, inflammatory, and neural systems [11,12,35]. This does not imply that HRV directly quantifies inflammation or oxidative stress. Rather, alterations in HRV may provide a non-invasive indication that the integrated regulatory environment has become less adaptable [11,12,35].

### 4.4. Mitochondrial Dysfunction and Metabolic Flexibility

Mitochondrial dysfunction represents another major component of the cardiometabolic phenotype [134]. Excess nutrient availability, ectopic lipid deposition, chronic inflammation, and oxidative stress can impair mitochondrial oxidative capacity and alter cellular energy metabolism [135,136]. These changes are particularly relevant to skeletal muscle and cardiovascular tissues because both require continuous and highly regulated ATP production [137].

Skeletal-muscle mitochondrial function is closely related to metabolic flexibility, defined broadly as the capacity to appropriately adjust substrate utilization according to physiological demand [138]. Impaired metabolic flexibility may contribute to insulin resistance, reduced exercise tolerance, and metabolic dysfunction [139,140]. Conversely, preservation or enhancement of skeletal muscle through physical activity may improve insulin sensitivity, mitochondrial function, substrate utilization, and inflammatory regulation [140].

Mitochondrial dysfunction may influence HRV indirectly through its effects on metabolic stress, endothelial function, inflammation, and cardiovascular demand [12,35,141,142]. Thus, reduced HRV should not be interpreted as a direct marker of mitochondrial impairment. Rather, HRV may reflect the integrated cardiovascular consequence of multiple metabolic processes in which mitochondrial dysfunction is an important component [12,35,141,142].

This distinction becomes particularly relevant when considering exercise adaptation. Exercise activates molecular pathways associated with mitochondrial remodeling and metabolic adaptation, including AMPK/PGC-1α signaling [23]. These pathways contribute to improved oxidative metabolism and metabolic efficiency and may ultimately influence cardiovascular autonomic regulation [143]. The potential relationship between mitochondrial adaptation and HRV therefore represents an important mechanistic bridge between molecular exercise responses and non-invasive physiological monitoring [11,12,35].

### 4.5. Endothelial Dysfunction and Baroreflex–Autonomic Interaction

Vascular dysfunction provides another important connection between cardiometabolic disease and HRV [144,145]. Insulin resistance, oxidative stress, inflammation, and hypertension can impair endothelial signaling, reduce nitric oxide bioavailability, and promote arterial stiffness and vascular remodeling. Because vascular function is closely integrated with autonomic regulation, these abnormalities may influence cardiovascular adaptability through altered baroreflex and mechanosensory signaling [146,147].

The baroreflex continuously detects changes in arterial pressure and adjusts sympathetic and parasympathetic output to preserve cardiovascular homeostasis [148]. Alterations in vascular compliance and arterial stiffness may modify the mechanical stimulus reaching arterial baroreceptors, potentially impairing this feedback system [149,150]. Baroreflex sensitivity (BRS) can be assessed non-invasively alongside HRV using sequence methods or cross-spectral analysis of continuous blood pressure and R–R interval recordings, providing complementary information on baroreflex–autonomic function [151]. At the same time, persistent sympathetic activation may contribute to vasoconstriction and vascular dysfunction, creating a reciprocal relationship between autonomic and vascular abnormalities [11,12,35].

Accordingly, reduced HRV in cardiometabolic disease may partly reflect impaired vascular–autonomic integration. This is particularly relevant in hypertension, metabolic syndrome, and obesity, where vascular dysfunction and autonomic abnormalities frequently coexist [16,66].

Exercise-induced vascular adaptations provide an important mechanistic counterpoint [152,153]. Increased vascular shear stress, improved nitric oxide bioavailability, reduced oxidative stress, and vascular remodeling are among the adaptations associated with regular physical activity [152,153]. Aerobic exercise may additionally activate angiogenic and mitochondrial pathways, including vascular endothelial growth factor signaling, thereby supporting vascular remodeling and tissue perfusion [152,153].

### 4.6. Skeletal Muscle as a Metabolic and Endocrine Regulator

Skeletal muscle occupies a central position within the cardiometabolic network because it simultaneously functions as a contractile tissue, a major site of glucose disposal, a metabolic organ, and an endocrine organ [154]. Muscle mass contributes to glucose homeostasis and energy expenditure, while muscle contraction stimulates the release of myokines that can influence distant tissues [155].

Loss of muscle mass or reduced muscle quality may therefore have consequences extending beyond physical performance [156]. Reduced muscle metabolic capacity may impair glucose disposal and lipid oxidation and contribute to systemic metabolic dysfunction. Conversely, preservation or enhancement of skeletal muscle may improve insulin sensitivity, mitochondrial function, metabolic flexibility, and inflammatory regulation [157,158].

The endocrine function of skeletal muscle provides an additional mechanism for inter-organ communication [23]. Contraction-induced myokines can influence adipose tissue, liver, pancreas, vasculature, and immune cells, creating a network through which exercise-induced muscle activity can modify systemic metabolic and inflammatory environments [23,89,99,159]. Among these myokines, irisin has been implicated in exercise-related adipose tissue browning and metabolic regulation, whereas exercise-induced interleukin-6 (IL-6) may contribute to metabolic adaptation and exert context-dependent anti-inflammatory effects. Brain-derived neurotrophic factor (BDNF), which can also be influenced by exercise, has been associated with metabolic and neuroautonomic adaptations and may contribute to the modulation of autonomic cardiovascular regulation [160]. These changes may subsequently influence autonomic cardiovascular regulation.

This framework expands the interpretation of HRV beyond a cardiac-centered biomarker [11,12,35]. Changes in HRV during or following exercise may reflect altered autonomic signaling, but they may also occur in parallel with improvements in muscle metabolism, reduced adipose dysfunction, enhanced vascular function, and modified inter-organ communication [11,12,35]. Thus, HRV may represent one physiological output of a broader multisystem adaptation [11,12,35].

### 4.7. Neuroendocrine Regulation and the Autonomic–Metabolic Feedback Loop

The neuroendocrine system provides a final regulatory layer connecting metabolic status with cardiovascular autonomic function [161]. Adipose tissue, skeletal muscle, the hypothalamus, endocrine organs, and the cardiovascular system communicate continuously through hormonal and neural signals involved in energy balance, stress responses, appetite, glucose regulation, and cardiovascular homeostasis [23].

Chronic metabolic stress can disrupt this network through altered adipokine signaling, insulin resistance, inflammatory activation, and persistent neuroendocrine stimulation [23]. In particular, activation of the hypothalamic–pituitary–adrenal (HPA) axis and sympathetic-adrenal-medullary drive, characterized by altered cortisol and catecholamine dynamics, represents an important neuroendocrine pathway linking metabolic stress with autonomic dysfunction. Persistent activation of these systems may increase sympathetic drive, impair parasympathetic flexibility, and contribute to reduced vagally mediated HRV [162]. These changes may increase sympathetic drive while reducing the flexibility of parasympathetic responses. HRV may consequently provide a dynamic physiological expression of these broader neuroendocrine alterations [67,161,163].

Importantly, the autonomic–metabolic relationship should be conceptualized as a feedback loop rather than a unidirectional pathway [164]. Metabolic dysfunction may impair autonomic regulation, whereas persistent autonomic dysregulation may contribute to altered glucose metabolism, vascular function, inflammatory signaling, and energy balance. Over time, these reciprocal interactions may progressively reduce physiological resilience [89,165].

This model may also explain why individuals with apparently similar anthropometric or metabolic profiles can exhibit markedly different HRV responses and different capacities to tolerate physiological stress [11,12,35]. Genetic and environmental factors, including physical activity, sedentary behavior, sleep, diet, and chronic stress, may further modify this interaction [166,167].

### 4.8. HRV as an Integrative Phenotype of Cardiometabolic Resilience

The mechanisms described above converge on a central concept: HRV may be better understood as an integrative physiological phenotype of cardiometabolic resilience than as an isolated marker of autonomic balance [11,12,35]. Excess visceral adiposity can promote inflammation, oxidative stress, insulin resistance, mitochondrial impairment, and endothelial dysfunction [101,102,103]. These processes interact with neuroendocrine and autonomic pathways and may progressively reduce cardiovascular adaptability and vagally mediated HRV [168].

This systems perspective also provides a mechanistic explanation for the substantial inter-individual variability observed in HRV. Individuals with similar BMI, glycemic status, or blood pressure may nevertheless differ in adipose distribution, skeletal-muscle mass, inflammatory status, physical fitness, sleep, stress exposure, and autonomic regulation [169,170,171]. HRV may therefore contribute information regarding the individual’s current physiological capacity to adapt to internal and external stressors that is not captured by conventional static biomarkers [11,12,35].

However, the interpretation of HRV must remain cautious. HRV is influenced by numerous physiological and behavioral factors, and a change in HRV cannot be attributed to a single molecular pathway [172]. Accordingly, its greatest value may lie in integration with complementary measures of body composition, glycemic control, blood pressure, cardiorespiratory fitness, inflammatory status, and functional capacity rather than in its isolated use [101,102].

This integrated view establishes the conceptual bridge to the clinical and translational potential of HRV [173]. If HRV reflects the cumulative interaction among autonomic, metabolic, vascular, inflammatory, muscular, and neuroendocrine systems, repeated assessment may provide a practical means of tracking physiological adaptation over time [11,12,35]. The next section therefore considers how this integrative biomarker may contribute to cardiometabolic risk characterization, treatment monitoring, and individualized exercise strategies [12,13,16,35,66].

## 5. HRV and Exercise

HRV provides a dynamic, non-invasive perspective on the autonomic regulation of the cardiovascular system during and after exercise. Unlike resting HRV assessment, the evaluation of HRV in the context of exercise can provide insight into the continuous autonomic adjustments elicited by changing physiological demands, as well as the recovery of autonomic function following physical exertion. Accordingly, HRV has been increasingly investigated as a useful tool for monitoring exercise responses, characterizing autonomic regulation across different exercise intensities, and assessing post-exercise recovery [64,174,175].

### 5.1. HRV and Physiological Thresholds During Incremental Exercise

Because cardiac autonomic regulation changes continuously with exercise intensity, HRV has been investigated as a non-invasive tool for characterizing metabolic transitions during incremental exercise. During progressive exercise, parasympathetic withdrawal occurs early, followed by progressively greater sympathetic activation as exercise intensity increases [176]. These autonomic transitions are accompanied by changes in metabolic substrate utilization, ventilation, lactate production, and cardiovascular demand [176]. Consequently, the nonlinear behavior of HRV during incremental exercise may provide information regarding transitions between physiological exercise domains [177,178].

A classical physiological framework divides incremental exercise into three broad phases. Phase I corresponds to predominantly aerobic metabolism, generally associated with the first metabolic threshold and relatively low blood lactate concentrations. Phase II represents the transition toward greater anaerobic contribution, with increasing lactate accumulation and progressively greater cardiovascular stress. Phase III corresponds to severe-intensity exercise characterized by substantial glycolytic contribution, metabolic instability, and progressive loss of physiological homeostasis [179,180,181,182].

HRV-derived measures may provide a non-invasive approximation of these transitions. Changes in RMSSD, SD1, and DFA α1 have been investigated as potential markers of alterations in autonomic complexity associated with increasing exercise intensity [178,183]. During progressive exercise, the progressive withdrawal of vagal modulation produces characteristic changes in vagally mediated HRV parameters. DFA α1 may additionally demonstrate a transition from more complex fractal dynamics toward more constrained patterns as exercise intensity increases [178].

This approach may be particularly useful in individuals for whom conventional exercise testing presents practical limitations. In populations with overweight or obesity, cycle-ergometer protocols may be advantageous because they can reduce biomechanical constraints and facilitate standardized incremental testing [184]. Nevertheless, HRV-derived metabolic thresholds should be interpreted as complementary physiological estimates rather than replacements for gold-standard metabolic measurements such as direct lactate or gas-exchange assessment [177].

**Figure 2:**
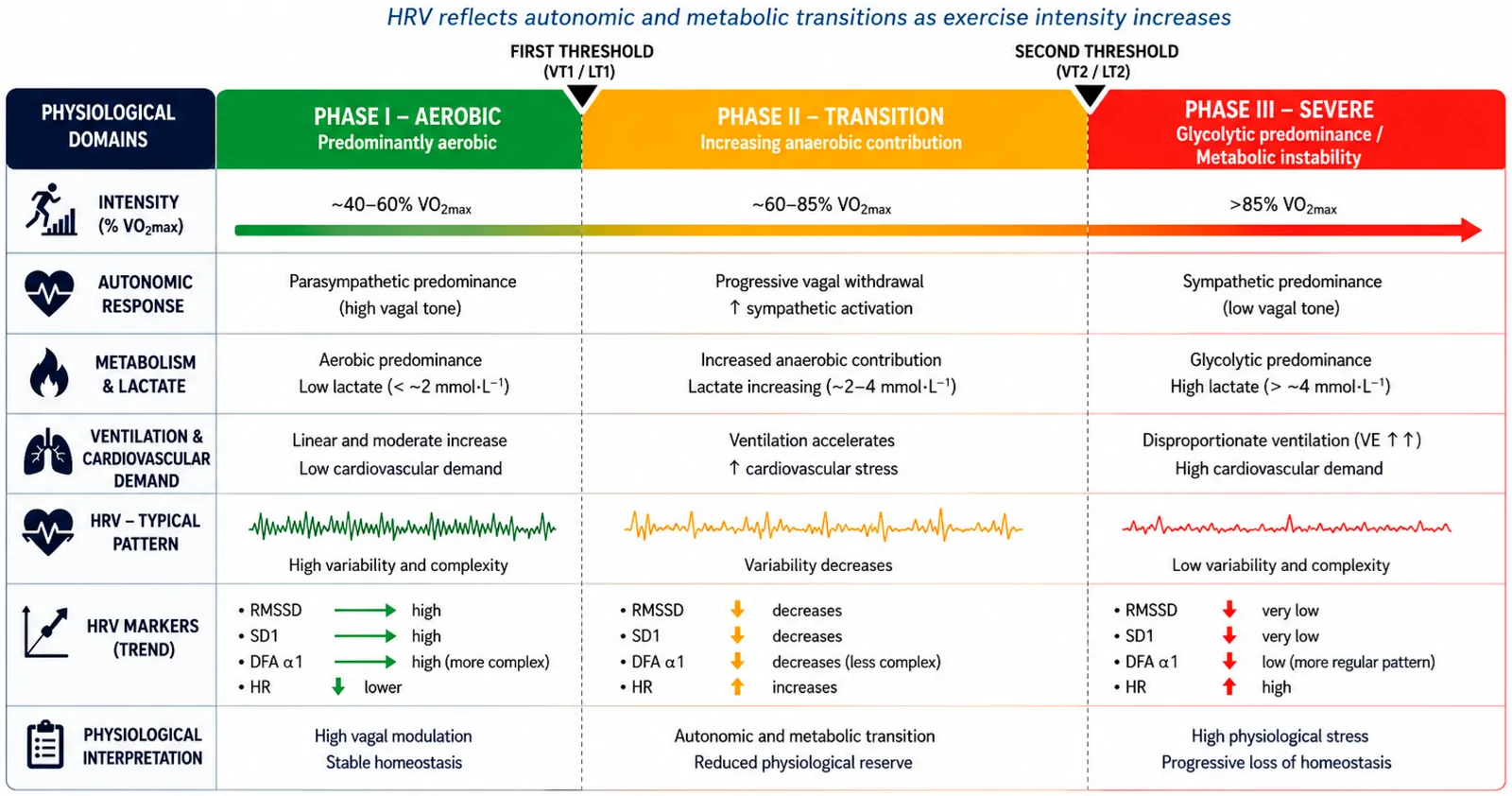
Physiological Thresholds and HRV during incremental exercise. DFA α1: Detrended Fluctuation Analysis α1, HR: Heart rate, HRV: Heart Rate Variability, RMSSD: Root Mean Square of the Successive Differences, SD1: Standard Deviation 1, VE: Minute ventilation, VT1/LT1: First ventilatory/lactate threshold, VT2/LT2: Second ventilatory/lactate threshold. Source: Elaboration owns.

Figure 2 summarizes the physiological responses occurring across incremental exercise domains and illustrates their relationship with changes in HRV-derived markers.

### 5.2. HRV and Post-Exercise Autonomic Recovery

The post-exercise period provides additional information regarding cardiovascular adaptability. Recovery can be broadly conceptualized as an early rapid phase followed by a slower phase extending from several hours to, depending on exercise load and individual characteristics, subsequent days. The initial phase is dominated by rapid parasympathetic reactivation and restoration of cardiovascular homeostasis, whereas the slower phase involves continued metabolic, hormonal, inflammatory, and autonomic recovery [185,186].

RMSSD and RR intervals are particularly useful for evaluating early vagal reactivation. A faster return toward baseline RMSSD generally indicates more efficient parasympathetic recovery, whereas delayed restoration may indicate greater physiological strain or incomplete recovery [186,187]. During prolonged recovery, complementary measures such as HF power, SD1/SD2, DFA α1, and other nonlinear indices may provide additional information regarding the restoration of autonomic organization [188].

The assessment of post-exercise HRV is especially relevant in cardiometabolic disease because impaired autonomic recovery may indicate reduced physiological resilience [11,35]. Individuals with obesity, insulin resistance, hypertension, or diabetes may exhibit altered autonomic responses to exercise, reflecting the combined influence of metabolic dysfunction, inflammation, vascular impairment, and reduced cardiorespiratory fitness [11,12,35].

**Figure 3:**
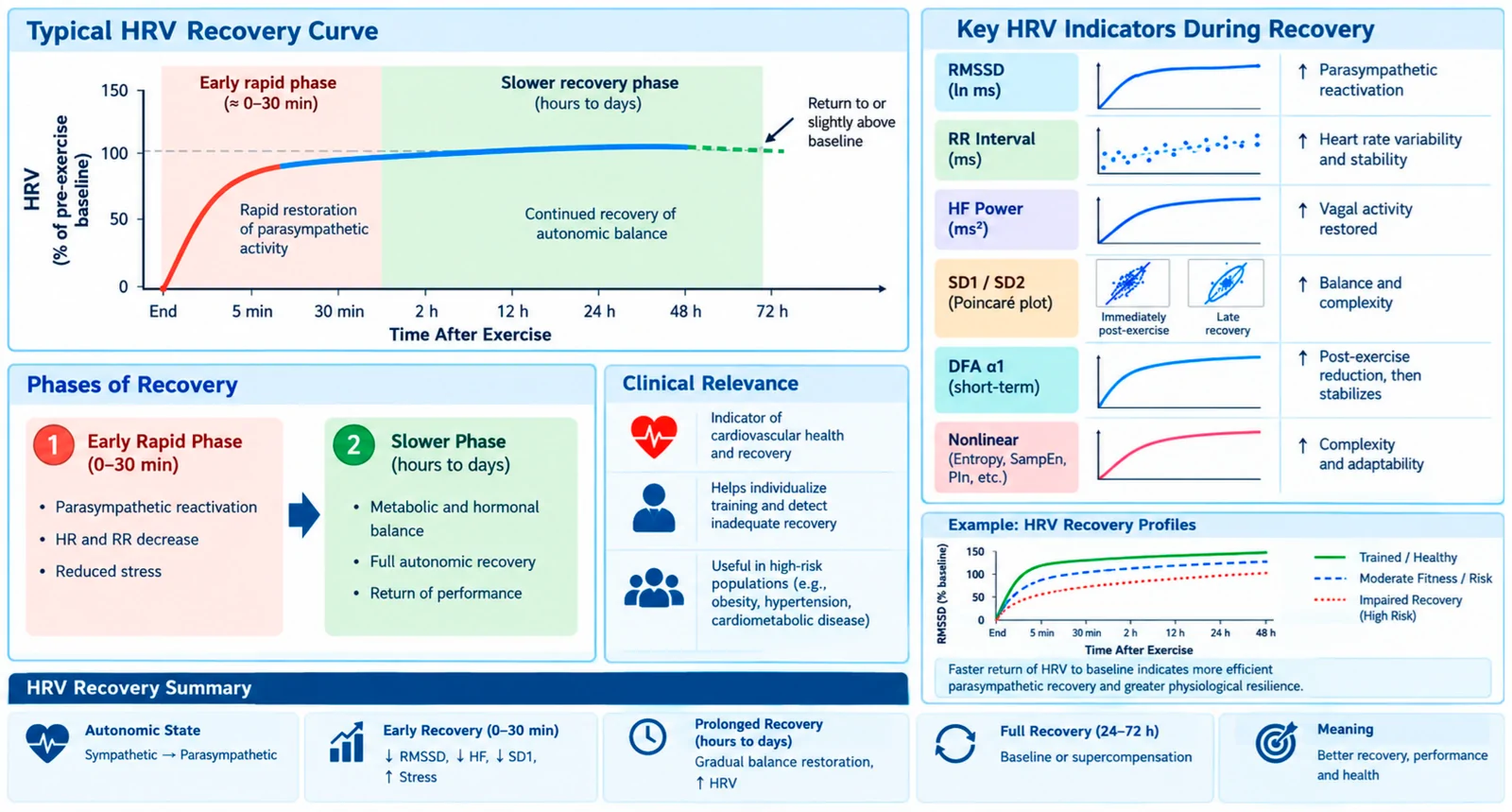
Post-exercise HRV dynamics. DFA α1: Detrended Fluctuation Analysis α1, HF: High Frequency, HRV: Heart Rate Variability, RMSSD: Root Mean Square of the Successive Differences, RR: R-R Interval (or Beat-to-Beat Interval), SD1: Standard Deviation 1, SD1/SD2: SD1 to SD2 Ratio. Source: Elaboration owns.

Figure 3 summarizes post-exercise HRV dynamics.

### 5.3. HRV, Cardiovagal Training, and Exercise Prescription

The concept of cardiovagal training has emerged from the recognition that exercise can induce favorable adaptations in parasympathetic regulation and cardiovascular recovery [11,12,35]. Aerobic exercise is particularly relevant because repeated cardiovascular stimulation can improve cardiorespiratory fitness, endothelial function, metabolic regulation, and autonomic recovery. Improvements in brain–heart communication and vagally mediated cardiovascular control have been described following structured aerobic training [31].

In populations with obesity, improvements in autonomic regulation may occur alongside favorable metabolic and hormonal adaptations. Exercise-induced reductions in leptin resistance and improvements in cardiovascular function have been associated with enhanced cardiometabolic health [189,190]. These adaptations are relevant because adipose tissue dysfunction, inflammation, insulin resistance, and autonomic impairment are mechanistically interconnected [23].

The prescription of cardiovagal-oriented exercise should consider the fundamental principles of training, including volume, intensity, frequency, and density, while recognizing substantial inter-individual variability in autonomic responsiveness [28,191]. From an evidence-based perspective, three distinct levels of evidence should be considered when interpreting the potential role of HRV in exercise prescription: (i) changes in HRV in response to exercise; (ii) the use of HRV as a marker of recovery or physiological readiness; and (iii) whether modifying exercise prescription according to HRV produces superior cardiometabolic outcomes. Although the first two levels are supported by an expanding body of evidence, evidence for the third remains limited, particularly in populations with cardiometabolic disease [11,173].

Accordingly, repeated HRV measurements may provide a practical means of monitoring individual physiological responses and informing exercise-load adjustments rather than serving as a stand-alone basis for exercise prescription [11,173]. This distinction is particularly important because evidence supporting HRV-guided training is currently stronger in athletic and endurance populations than in patients with cardiometabolic disease.

This approach is particularly relevant to personalized exercise medicine [11,173]. HRV can be integrated with cardiorespiratory fitness, blood pressure, metabolic biomarkers, body composition, sleep, perceived recovery, and clinical status to characterize an individual’s physiological phenotype [11,12,35]. Such multimodal integration may help determine whether an individual appears adequately recovered to tolerate a given exercise stimulus or whether temporary modification of intensity, volume, or recovery duration could be considered [192]. However, HRV-derived information should be interpreted as complementary to, rather than a replacement for, established clinical and functional assessments.

From this perspective, HRV should not be considered simply an outcome variable. It may also function as a dynamic feedback signal within an adaptive exercise-prescription model. Repeated monitoring can provide information on the relationship between training load, autonomic response, and recovery, potentially supporting individualized adjustments in subsequent exercise progression [64,174,175]. Nevertheless, whether such HRV-informed adjustments consistently translate into superior cardiometabolic adaptations remains insufficiently established.

Overall, the physiological framework presented in this section establishes HRV as a multidimensional, non-invasive physiological signal situated at the intersection of autonomic regulation, cardiovascular physiology, metabolic demand, and recovery [64,173,174]. Its interpretation becomes particularly valuable when integrated with the molecular mechanisms underlying cardiometabolic disease [153]. These mechanisms—including insulin resistance, chronic inflammation, oxidative stress, mitochondrial dysfunction, endothelial impairment, and neuroendocrine dysregulation—may influence autonomic regulation and consequently become partially expressed through alterations in HRV [153]. This provides a conceptual basis for examining HRV as an integrative physiological marker in cardiometabolic disease and for exploring its potential role in personalized exercise therapy [11,12,35]. However, HRV-guided exercise prescription in cardiometabolic populations should currently be regarded as a promising strategy rather than an established clinical approach, pending stronger evidence demonstrating that HRV-based prescription improves clinically relevant cardiometabolic outcomes.

**Figure 4:**
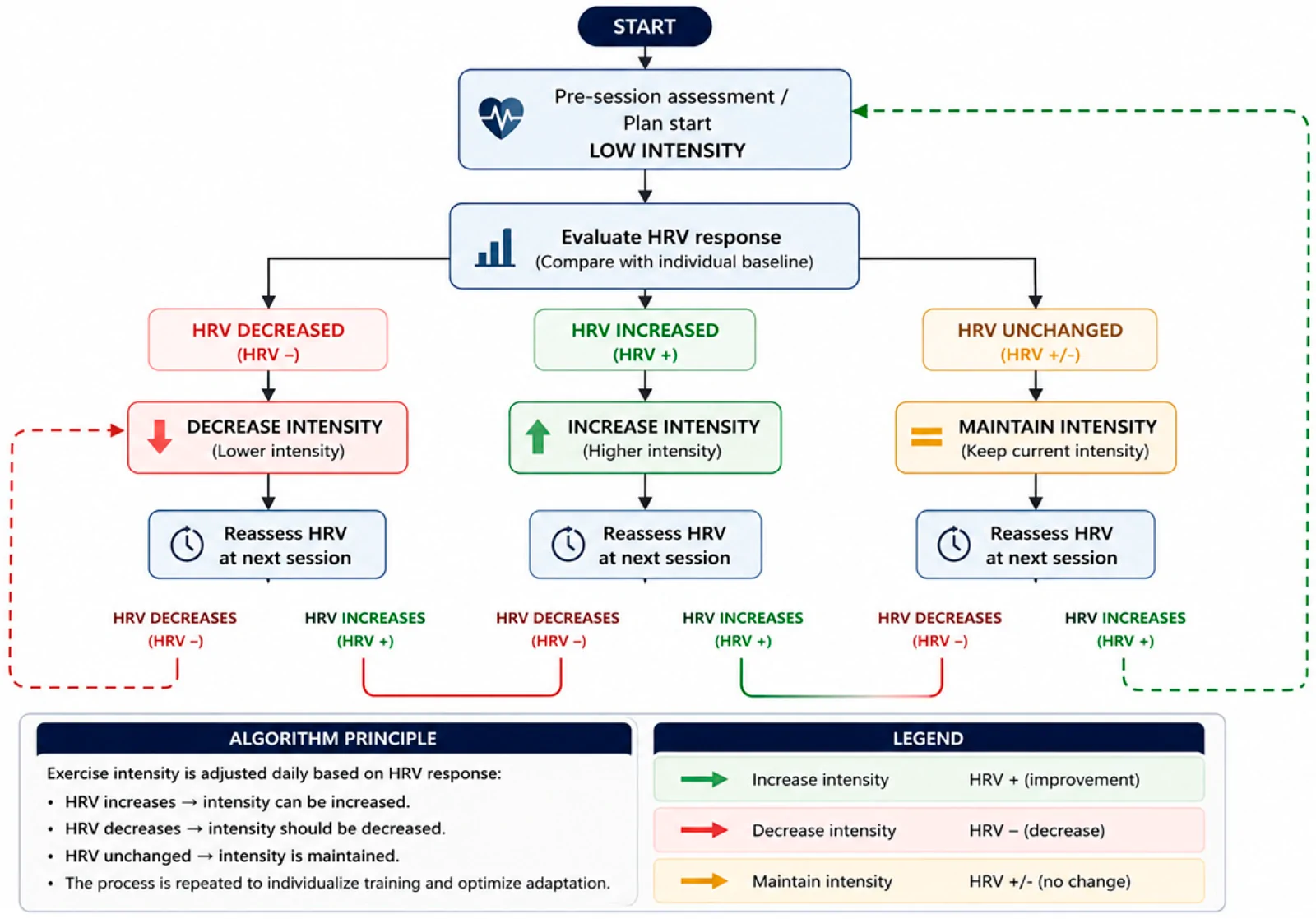
Algorithm for prescribing training loads using HRV. HRV: Heart Rate Variability. Source: adaptation of Casanova-Lizón et al. [193].

Figure 4 summarizes the algorithm for exercise prescription based on HRV.

## 6. Clinical and Translational Implications

The potential clinical value of HRV lies in its ability to provide non-invasive, inexpensive, repeatable information regarding physiological regulation [167]. Unlike many conventional biomarkers that require blood sampling or specialized equipment, HRV can be obtained through ECG-based systems and, under appropriate conditions, validated wearable technologies [37,38,39,41,52].

In cardiometabolic disease, HRV could potentially contribute to three complementary domains: risk stratification, treatment monitoring, and exercise personalization [28]. However, these applications are supported by different levels of evidence and should therefore be distinguished according to their current degree of clinical maturity [153,194].

First, reduced HRV may identify individuals with autonomic dysfunction who are at increased cardiometabolic risk. Evidence linking low HRV with mortality and cardiovascular events supports its prognostic relevance [11,12,14,16,17,19,35]. Nevertheless, HRV should not currently replace established clinical risk scores. Thus, the use of HRV for risk stratification has a relatively stronger evidence base than its use for treatment monitoring or individualized exercise prescription, although HRV should currently be considered a complementary prognostic measure rather than an independent clinical decision-making tool [153,194].

Second, HRV may be useful for monitoring treatment response. Improvements following exercise, weight loss, improved glycemic control, or cardiovascular rehabilitation may indicate enhanced autonomic adaptability. Systematic reviews have reported improvements in HRV following exercise interventions in type 2 diabetes and hypertension populations [26,123,124]. Nevertheless, changes in HRV should not be interpreted as a direct measure of treatment efficacy or as evidence of improvement in a specific cardiometabolic pathway, given the multifactorial determinants of HRV. Accordingly, treatment monitoring using HRV remains a potentially useful complementary application rather than an established stand-alone clinical approach [153,194].

Third, HRV may contribute to personalized exercise prescription. This application is particularly attractive because cardiometabolic disease is heterogeneous. Two individuals with similar BMI or HbA1c may have substantially different autonomic, inflammatory, cardiovascular, and functional profiles [83,84,112,169,170,171]. However, the evidence supporting HRV-informed modification of exercise prescription is currently less established than the prognostic evidence linking HRV with cardiometabolic risk. In particular, evidence that HRV-guided exercise prescription produces superior cardiometabolic outcomes remains limited, especially in clinical cardiometabolic populations [153,194].

A future precision-exercise framework could therefore stratify individuals according to metabolic and autonomic phenotype. Individuals with pronounced obesity and impaired cardiorespiratory fitness may require greater emphasis on aerobic training, whereas individuals with sarcopenic obesity or low muscle mass may require a greater resistance-training component. Patients with adequate fitness but poor autonomic recovery may benefit from greater attention to training load, recovery, sleep, and stress management [194,195]. At present, however, these applications should be regarded primarily as a future precision-exercise framework rather than as an established clinical algorithm. HRV may provide complementary information to support individualized decisions, but objective HRV monitoring should always be integrated with established clinical variables, including glycemic status, blood pressure response, and subjective fatigue, when adjusting exercise intensity and training load. Such decisions should remain integrated with established clinical, metabolic, functional, and exercise-related assessments [153,194].

Importantly, HRV should be considered an adjunctive biomarker. It is influenced by numerous physiological and behavioral factors, and changes in HRV do not necessarily indicate improvement or deterioration in one specific disease pathway. Its clinical applicability may also be limited in specific circumstances in which HRV cannot be reliably measured or interpreted [153,194]. For example, cardiac rhythm disturbances such as atrial fibrillation, frequent ectopic beats, or paced rhythms may compromise conventional R–R interval-based HRV indices, particularly when substantial beat-to-beat irregularity requires artifact correction or interpolation [196,197]. Acute physiological conditions, including fever, pain, recent strenuous exercise, dehydration, acute psychological stress, and marked sleep disruption, may also produce transient autonomic changes that do not necessarily reflect the patient’s stable cardiometabolic state [198,199,200]. In addition, medications that affect autonomic or cardiovascular function, including beta-blockers, antiarrhythmic agents, and some antihypertensive treatments, may alter HRV independently of changes in the underlying disease [201,202]. Therefore, medication status, acute clinical conditions, and measurement circumstances should be considered when interpreting HRV, particularly during longitudinal follow-up [58].

These limitations do not necessarily preclude HRV assessment but emphasize that measurements should preferably be obtained under standardized and clinically stable conditions and interpreted alongside other clinical, metabolic, functional, and cardiovascular information [58,153,194]. This is particularly important when small changes in HRV are used to inform individual clinical decisions, as physiological or methodological variability may otherwise be mistaken for meaningful changes in autonomic regulation [58,153,194]. Therefore, the translational potential of HRV should be interpreted according to the specific application: prognostic risk stratification is relatively well supported; treatment monitoring is promising but less established; and HRV-guided exercise personalization remains an emerging and investigational approach. This distinction is important to avoid overstating the current clinical utility of HRV while recognizing its potential role in future precision cardiometabolic care (Table 4).

## 7. Methodological Challenges and Future Directions

Despite the growing evidence supporting HRV as a cardiometabolic biomarker, several limitations currently restrict its clinical implementation.

First, HRV methodology remains heterogeneous. Studies differ in recording duration, posture, breathing conditions, time of day, ECG acquisition, preprocessing, artifact correction, and analytical methods. This heterogeneity makes direct comparison between studies difficult. Differences in these conditions can produce meaningful changes in HRV independently of the underlying physiological state, making it difficult to determine whether differences between studies reflect true differences in autonomic regulation or simply differences in measurement procedures. This issue is particularly relevant for commonly used short-term indices such as RMSSD, which may be sensitive to recording conditions and respiratory influences. Consequently, comparisons of absolute HRV values across studies should be interpreted cautiously unless acquisition and processing conditions are sufficiently comparable.

Standardization is also essential when HRV is used for longitudinal monitoring within an individual. For repeated assessments, measurements should ideally be obtained under comparable conditions, including the time of day, body position, recording duration, pre-measurement activity, environmental conditions, and, when possible, breathing pattern. Changes in HRV observed under substantially different measurement conditions may otherwise be misinterpreted as physiological adaptations or deterioration. Thus, individualized longitudinal monitoring should prioritize the magnitude and consistency of changes obtained under standardized conditions rather than relying exclusively on comparisons with population-based reference values.

An additional consideration is the distinction between HRV derived from ECG and measurements obtained from consumer wearable devices. ECG directly records cardiac electrical activity and therefore provides the conventional reference for identifying successive R–R intervals, whereas PPG-based devices estimate pulse-to-pulse intervals from peripheral blood-volume changes. Although PPG can provide acceptable estimates of HRV under resting and controlled conditions, its accuracy may be affected by motion, peripheral perfusion, sensor placement, signal quality, and device-specific algorithms. Accordingly, HRV values obtained from different devices should not automatically be considered interchangeable, particularly when small longitudinal changes are interpreted at the individual level. Validation against ECG and reporting of device characteristics and signal-processing procedures are therefore important for clinical and research applications.

Beyond these methodological considerations, the current clinical applications of HRV in specific cardiometabolic disorders should also be considered. In hypertension, reduced HRV and altered autonomic regulation have been associated with greater cardiovascular risk and may provide complementary information for risk stratification when interpreted alongside blood pressure and other established cardiovascular risk factors. In diabetes, autonomic dysfunction reflected by reduced HRV has been associated with disease duration, glycemic dysregulation, and cardiovascular autonomic neuropathy, suggesting potential utility for identifying patients at increased cardiovascular and autonomic risk. In obesity, lower HRV has been associated with metabolic dysfunction and impaired autonomic regulation, supporting its potential role in characterizing cardiometabolic risk and monitoring changes associated with weight management and exercise interventions.

From a prognostic perspective, reduced HRV and abnormal autonomic profiles have been associated with adverse cardiovascular outcomes across several cardiometabolic populations. However, HRV should currently be regarded as a complementary rather than a standalone prognostic marker, because its incremental predictive value beyond established clinical variables remains incompletely defined. Similarly, although changes in HRV may provide information about autonomic responses to lifestyle and exercise interventions, current evidence is insufficient to support HRV as a universal therapeutic decision-making tool. Its most plausible clinical role is therefore as an adjunctive biomarker for risk characterization, longitudinal monitoring, and individualized exercise prescription, rather than as a replacement for established diagnostic, prognostic, or therapeutic criteria.

Second, the physiological interpretation of individual HRV metrics remains complex. In particular, LF and LF/HF should not be interpreted simplistically as direct measures of sympathetic activity or sympathovagal balance. Greater emphasis should be placed on validated time-domain measures, particularly RMSSD, together with complementary indices and standardized physiological conditions.

Third, wearable devices introduce additional methodological challenges. Although PPG-based systems can provide valid HRV estimates at rest, movement artifacts and differences in algorithms may influence accuracy. This limitation may be particularly relevant in patients with cardiometabolic diseases, such as severe obesity, long-standing diabetes, or metabolic syndrome, who may present cardiac ectopy or clinically silent atrial fibrillation. Under these conditions, automated HRV algorithms may misidentify or exclude abnormal beats, generating artifacts and reducing the reliability of PPG-derived HRV estimates. Therefore, underlying arrhythmias and ectopic beats should be considered when interpreting HRV obtained from PPG-based wearable devices in cardiometabolic populations. The increasing availability of consumer devices therefore creates both an opportunity and a methodological challenge.

Fourth, reference values for HRV remain dependent on age, sex, fitness, posture, circadian phase, medication, and recording conditions. Individualized longitudinal monitoring may therefore be more informative than comparison against a single universal reference value.

Fifth, the causal direction of the relationship between HRV and cardiometabolic disease remains incompletely established. Reduced HRV may reflect disease burden, contribute to disease progression, or represent a combination of both. Longitudinal studies are required to determine whether changes in HRV independently predict future metabolic deterioration.

Future research should prioritize standardized protocols, repeated longitudinal measurements, and integration of HRV with molecular and clinical biomarkers. Multi-omics approaches incorporating transcriptomics, proteomics, metabolomics, epigenomics, and microbiome profiling may help identify biological phenotypes associated with autonomic dysfunction and exercise responsiveness.

Artificial intelligence and machine learning may further facilitate the integration of high-frequency wearable data with clinical and molecular information. However, predictive performance should be evaluated prospectively and externally validated before clinical implementation.

An important research direction is the identification of HRV phenotypes that predict response to specific exercise modalities. Rather than asking whether exercise generally increases HRV, future studies should determine which individuals respond to aerobic training, resistance training, HIIT, combined training, or HRV-guided approaches, and why.

Such research could ultimately establish a precision exercise model in which autonomic, metabolic, inflammatory, and molecular information is used to determine the optimal dose and modality of exercise for each patient.

## 8. Conclusions

HRV represents a promising non-invasive and dynamic biomarker of cardiovascular autonomic regulation in cardiometabolic disease. Rather than reflecting a simple sympathovagal balance, HRV may capture the integrated effects of metabolic, inflammatory, mitochondrial, vascular, endocrine, and autonomic dysfunction. Its dynamic response to exercise and recovery further supports its potential to characterize physiological adaptability and cardiometabolic resilience.

The integration of HRV with clinical, metabolic, functional, and body-composition measures may therefore contribute to more individualized monitoring and exercise prescription. However, methodological heterogeneity, physiological confounding, and the absence of definitive evidence establishing causality between HRV alterations and cardiometabolic disease remain important limitations. Future longitudinal and mechanistic studies should determine whether specific HRV phenotypes can reliably guide personalized exercise strategies and improve cardiometabolic outcomes.

## Figures and Tables

**Table 1:** Principal HRV Parameters in Time Domain.

HRV Parameter	Principal Physiological Information	Potential Relevance
RR	Mean duration of normal-to-normal RR intervals	Provides the average cardiac cycle and contextualizes HRV measures; influenced by resting heart rate and autonomic state
SDNN	Overall RR interval variability	Global autonomic/cardiovascular regulation
SDANN	Standard deviation of the average NN intervals across recording segments	Reflects long-term fluctuations in HRV and circadian/autonomic regulation
RMSSD	Short-term beat-to-beat variability	Vagal-related modulation
NN50	Number of adjacent NN intervals differing by >50 ms	Index of short-term beat-to-beat variability with predominantly vagal influence
pNN50	Percentage of successive NN intervals that differ by >50 ms	Parasympathetic-related modulation

HRV: Heart Rate Variability; NN50: Number of pairs of successive NN intervals that differ by more than 50 milliseconds; pNN50: Proportion of NN50 divided by the total number of NN intervals; RMSSD: Root Mean Square of Successive Differences; RR: R-R Interval; SDNN: Standard Deviation of NN intervals. Source: Adaptation of Shaffer et al. [42] and Billman et al. [67].

**Table 2:** Principal HRV Parameters in Frequency Domain.

HRV Parameter	Principal Physiological Information	Potential Relevance
Total power	Overall spectral power across the analyzed frequency range	Global representation of oscillatory cardiovascular regulation; highly dependent on recording duration
ULF power	Power in the ultra-low-frequency range	Long-term regulatory influences, including circadian and systemic processes; mainly relevant to 24-h recordings
VLF power	Very-low-frequency oscillatory power	Associated with multiple slow regulatory processes; interpretation should be cautious, particularly in short recordings
LF power	Power in the low-frequency range (0.04–0.15 Hz), reflecting mixed cardiovascular/autonomic regulation and baroreflex modulation.	Baroreflex and autonomic modulation
LFnu	LF power expressed in normalized units	Relative contribution of LF oscillations after accounting for VLF components; should be interpreted as mixed autonomic modulation rather than sympathetic tone
HF power	Respiratory-related oscillations	Predominantly vagal modulation
HFnu	HF power expressed in normalized units	Relative contribution of respiratory-related vagal modulation
LF/HF ratio	Ratio between LF and HF components	Should be interpreted cautiously; not a direct sympathovagal index

HRV: Heart Rate Variability; HF power: High Frequency power; HFnu: High Frequency normalized units; LF power: Low Frequency power; LF/HF ratio: Ratio of Low Frequency to High Frequency power; LFnu: Low Frequency normalized units; ULF power: Ultra Low Frequency power; VLF power: Very Low Frequency power. Source: Adaptation of Shaffer et al. [42] and Billman et al. [67].

**Table 3:** Principal HRV Parameters in Non-linear Domain.

HRV Parameter	Principal Physiological Information	Potential Relevance
SD1	Short-axis dispersion of successive RR intervals	Short-term beat-to-beat variability, closely related to RMSSD and vagal modulation
SD2	Long-axis dispersion of RR intervals	Longer-term variability and global dynamics of the RR interval series
SD1/SD2	Short- and long-term Poincaré dynamics	Complementary characterization of HRV
DFA α1	Short-term fractal scaling properties of RR intervals	Reflects short-range organization and dynamic adaptability of cardiovascular regulation
DFA α2	Long-term fractal scaling properties of RR intervals	Characterizes longer-range organization of cardiovascular dynamics
ApEn	Regularity and predictability of the RR interval series; includes self-matching, making it sensitive to data length	Provides information on cardiovascular complexity and adaptability
SampEn	Complexity and irregularity of the RR interval signal; excludes self-matching, reducing bias and dependence on data length	Potential marker of altered cardiovascular regulatory complexity

HRV: Heart Rate Variability, ApEn: Approximate Entropy; DFA α1: Detrended Fluctuation Analysis α1; DFA α2: Detrended Fluctuation Analysis α2; SampEn: Sample Entropy; SD1: Standard Deviation 1; SD1/SD2: Ratio of SD1 to SD2; SD2: Standard Deviation 2. Source: Adaptation of Shaffer et al. [42] and Billman et al. [67].

**Table 4:** Summarizes the current evidence, principal limitations, and clinical status of the three proposed applications of HRV in cardiometabolic disease.

Proposed Application	Current Evidence	Main Limitations	Clinical Status	References
Risk stratification	Relatively strong prognostic evidence linking reduced HRV with cardiovascular events and mortality	Influenced by age, medications, comorbidities, physical fitness, measurement conditions, and other physiological factors	Complementary Clinical prognostic application; should not replace established risk scores	[11,12,14,16,17,19,35,153,194,198,200]
Treatment monitoring	Evidence of HRV Improvements Following exercise and improvements in metabolic or cardiovascular status	Changes in HRV are nonspecific and do not necessarily reflect improvement in particular cardiometabolic pathway	Promising but not established as a stand-alone monitoring tool	[26,58,123,124,153,194]
Exercise personalization	Growing evidence supporting HRV monitoring for physiological recovery and adaptation, but limited evidence that HRV-guided prescription improves cardiometabolic outcomes	Limited clinical evidence; stronger evidence currently comes from athletics populations; substantial inter-individual and methodological variability	Emerging investigational approach rather than established clinical practice	[29,51,58,74,153,174,175,188,189,194]

## Data Availability

No new data were created or analyzed in this study. Data sharing is not applicable to this article.
